# Enhanced voltage generation in microbial fuel cells (MFCs) using bacterial isolates from seawater and industrial wastewater

**DOI:** 10.1186/s12934-025-02892-w

**Published:** 2025-12-28

**Authors:** Ghada E. Hegazy, Nadia A. Soliman, Yasser R. Abdel-Fattah, Tarek H. Taha

**Affiliations:** 1 National Institute of Oceanography & Fisheries, NIOF-Egypt, Alexandria, Egypt; 2 Bioprocess Development Department, Genetic Engineering & Biotechnology Research Institute (GEBRI), City of Scientific Research & Technological Applications, Alexandria, Egypt; 3 Environmental Biotechnology Department, Genetic Engineering and Biotechnology Research Institute (GEBRI), City of Scientific Research and Technological Applications, Alexandria, Egypt

**Keywords:** Microbial fuel cell, Wastewater treatment, Electrogenic bacteria, Renewable energy, Sustainable technology, Bioelectricity

## Abstract

**Background:**

This study investigates the potential of microbial fuel cells (MFCs) for bioelectricity generation from seawater and wastewater sources. It focuses on the isolation, identification, and statistical optimization of electrogenic bacteria from diverse environmental samples, aiming to enhance sustainable production of bioenergy.

**Results:**

Four bacterial isolates were obtained from Max surface water, oil factory wastewater, Abu-Qir bottom sediment, and fish factory wastewater. *16 S rRNA* gene sequencing identified these isolates as *Stenotrophomonas* sp. strain S2 (El-Max), *Bacillus paralicheniformis* strain O3 (Oil factory), *Bacillus safensis* strain QB (Abu-Qir), and *Serratia* sp.strain GH3 (Fish factory). Initial screening of microbial consortia showed promising bioelectricity generation, with voltage outputs ranging from 0.175 V to 0.542 V crosswise isolates. Statistical method using Plackett–Burman Design (PBD) screened the key factors influencing voltage production, including pH, time, oxygen, inoculum size, mediator, and resistance. Each isolate exhibited a distinct pattern of factor significance, yet the models for the four strains demonstrated excellent predictive power with *R²* values near 0.98 or higher.

**Conclusion:**

These results underscore the strong potential of specific electrogenic bacterial strains, isolated from diverse wastewater sources, to enhance bioelectricity production in Microbial Fuel Cells (MFCs). The identification of critical operational parameters provides valuable insights for optimizing MFC performance. Together, these findings demonstrate the viability of MFCs as an effective dual-purpose technology for wastewater treatment and renewable energy production.

**Supplementary Information:**

The online version contains supplementary material available at 10.1186/s12934-025-02892-w.

## Introduction

The world’s growing need for energy is undeniable, but the way we currently get most of that energy – by burning fossil fuels – is causing significant harm to our planet [1]. Obvious increasing signs of climate changes have been noticed, including rising temperatures, more extreme weather events, and rising sea levels [2]. Air and water pollution, also largely caused by fossil fuel use, threaten human health and damage ecosystems [3]. At the same time, ever-increasing amounts of wastewater are generated from factories, farms, and homes. This wastewater contains pollutants that can harm rivers, lakes, and oceans if not treated properly. Therefore, it is urgently needed to discover and implement cleaner, greener ways to generate energy and effectively treat wastewater, simultaneously addressing both critical environmental challenges [4]. Microbial fuel cells (MFCs) are emerging as a promising and innovative technology that has the potential to help solving both of these pressing problems. Unlike traditional energy sources that rely on combustion or non-renewable resources, MFCs harness the power of tiny living organisms, called microorganisms, to break down organic waste materials and directly generate electricity. This process is essentially a bio-catalyzed conversion, mimicking natural processes that occur in the environment. It is like copying what happens in nature, where bacteria decompose organic matter and release energy in the process. Instead of simply releasing that energy as heat, MFCs capture the released electrons and channel them to create an electrical current. This ability to convert waste into energy makes MFCs a potentially game-changing technology for sustainable energy production and wastewater treatment [5, 6]. MFCs offer a range of compelling advantages compared to traditional methods of energy generation and wastewater treatment. First and foremost, they use waste materials as fuel, which are abundant and renewable resources. Instead of relying on finite fossil fuels, MFCs can utilize organic matter present in wastewater, agricultural runoff, and other waste streams, effectively turning a problem into a resource [7]. Second, MFCs operate at relatively low temperatures and pressures, requiring significantly less energy input compared to conventional treatment methods that often involve high-energy processes like aeration and chemical addition. This lower energy footprint contributes to a more sustainable and environmentally friendly overall process [8]. Third, MFCs have the potential to significantly reduce pollution and greenhouse gas emissions. By converting organic waste into energy, they reduce the need for landfills and incineration, which release harmful pollutants into the environment [9]. Finally, MFCs can be designed and deployed in small, decentralized settings, offering a flexible and adaptable solution for providing energy and treatment to communities that may not have access to centralized infrastructure [10]. Despite their immense potential, MFCs still face several significant challenges that hinder their widespread adoption and commercialization. One of the primary challenges is their relatively low power output compared to established energy sources like solar or wind power. While MFC technology is constantly improving, the amount of electricity generated per unit volume is still a limiting factor for many applications [11]. Another challenge is the cost of construction and maintenance of MFC systems. The materials used to build MFCs, such as electrodes and membranes, can be expensive, and the long-term stability and performance of these components are still being investigated [12]. A third challenge is the need to identify and engineer more efficient microorganisms that are capable of rapidly breaking down organic matter and effectively transferring electrons to the electrode. This requires a deeper understanding of the complex interactions between microorganisms and the electrode surface [13]. Finally, scaling up MFC technology from small laboratory experiments to large-scale working systems presents significant engineering and economic hurdles [14]. To overcome these limitations, contemporary MFC research is rapidly advancing through interdisciplinary approaches. Specifically, nanotechnology is being leveraged for enhanced electron transfer at the microbe-electrode interface, and the development of functional energy materials is leading to drastically improved electrode performance and longevity [12]. Furthermore, deep learning and other artificial intelligence (AI) techniques are increasingly being employed for real-time MFC optimization and predictive performance modeling, moving beyond traditional trial-and-error methods [14]. These technological leaps are also driving expanded applications in fields such as highly sensitive biosensors, efficient biomass conversion, and ultimately, large-scale, scaled-up wastewater treatment facilities [14]. The microorganisms are arguably the single most critical component of MFCs, as they are the engines that drive the entire process. These tiny organisms act as biocatalysts, breaking down complex organic molecules in the waste stream and releasing electrons in the process. These electrons are then captured by the electrode and flow through an external circuit, generating electricity. The efficiency of this process, particularly how well the microorganisms transfer the electrons to the electrode, is paramount to the overall performance of the MFC. Therefore, identifying, isolating, and studying microorganisms that are particularly adept at producing and transferring electrons is crucial for improving MFC technology. The selection of these microorganisms may improve the overall sustainability for electric generation on bacterial means [15]. The composition of the microbial community within an MFC can also have a profound impact on its performance. MFCs can be inoculated with mixed cultures of microorganisms, representing a diverse community of bacteria, or with pure cultures of a single, well-defined microorganism. Using a mix of different microorganisms can be advantageous because different species may be capable of breaking down different types of organic matter, leading to a more complete and efficient degradation of the waste stream. Moreover, a diverse microbial community can be more resilient to changes in the environmental conditions, making the MFC more stable over time [15]. On the other hand, using a pure culture allows researchers to study the specific metabolic pathways and electron transfer mechanisms of a single, well-characterized microorganism. This can provide valuable insights into how to optimize the performance of that particular species [15]. Researchers also actively search for and isolate microorganisms from diverse environments that are naturally efficient at generating electricity. To further enhance the performance of MFCs, researchers can manipulate a variety of factors that influence the growth and activity of the microorganisms. These factors include the type of organic matter provided as food for the microorganisms, the pH level within the MFC, the operating temperature, the materials used to construct the electrodes, the number of microorganisms used to inoculate the MFC, and the addition of special chemicals called mediators that can facilitate electron transfer. By carefully controlling and optimizing these parameters, it is possible to significantly increase the amount of electricity produced by the MFC [11]. To efficiently explore the complex interactions between these various factors, researchers often employ statistical experimental design methods, such as Plackett-Burman Design (PBD). PBD allows the systematic evaluation of multiple factors simultaneously, identifying which factors have the greatest impact on MFC performance and guiding further optimization efforts [16]. Razzak et al. [17] demonstrated that integrating microalgae with MFCs in a constructed wetland setup significantly boosted open-circuit voltage (up to ~ 786 mV) and power density (~ 50 mW/m²) [17]. Also recent research has expanded the range of known electrogenic microorganisms in microbial fuel cells (MFCs) beyond classical strains like *Geobacte*r and *Shewanella* to include non-traditional electrogens such as *Bacillus altitudinis* and native microbial communities from diverse environments, which show promising current generation capabilities [18]. Our current study aligns precisely with these modern trends toward efficient, cost-effective, and eco-friendly MFC designs. By focusing on the isolation and identification of novel, highly-active microbial strains from diverse, real-world wastewater environments and by using Plackett-Burman Design (PBD) for systematic operational optimization, we directly address the fundamental biological and engineering bottlenecks that hinder wide-scale MFC application. This study aims to advance MFC technology by investigating microorganisms sourced from four distinct types of wastewater and seawater: harbor water (El-Max), oil-contaminated wastewater from oil factory, sediment from Abu-Qir, and effluent from a fish processing facility. The specific objectives are: (1) to isolate and identify microbial strains from these environments with the potential to generate electricity within MFC systems; (2) to evaluate the electrochemical performance of these strains, focusing on their capacity for power generation and organic matter degradation; and (3) to optimize operational parameters for each strain using Plackett–Burman design (PBD), thereby enhancing MFC efficiency. The ultimate goal is to isolate four distinct bacterial strains, each exhibiting unique electroactive properties and enhanced power output under tailored PBD-optimized conditions that account for strain-specific resistance factors. Through this research, we aim to contribute to the development of more efficient and sustainable MFCs capable of converting wastewater into a renewable source of clean energy. In the long term, we envision MFC technology is playing a key role in supporting circular economy models by simultaneously addressing energy and environmental challenges on a global scale.

## Materials and methods

### Sample collection

Under aseptic conditions, surface and bottom seawater samples (50 m depth) were collected in sterile 500 mL bottles from Abu Qir and El-Max areas in Alexandria. Waste samples were also collected from factories in the Borg El-Arab industrial zone (Alexandria). These included fish waste, collected from the drainage outlet of a fish processing factory after final processing steps, and oil waste, collected from the drainage outlet of an edible oil extraction factory. All samples were immediately transported to the microbiology lab and stored at 4 °C until analysis [19].

### Physical parameter analysis

Temperature, pH, salinity, and conductivity were measured in all water samples using calibrated portable instruments: a digital thermometer, pH meter, refractometer, and conductivity meter, respectively. All measurements were taken immediately after sample collection and recorded in 25 °C, unitless pH, ppt, and µS/cm, respectively.

### Dissolved oxygen (DO) determination

Dissolved oxygen was measured using the Winkler titration method [20]. Reagents 1 (400 g MnCl_2_·4H_2_O + 2 ml conc. HCl/L) and 2 (360 g NaOH + 400 g KI/L) were added (1 ml each) to 100 ml of each water sample. After precipitate formation, 1 ml conc. H₂SO₄ was added to dissolve it. A 50 ml aliquot was titrated with 0.02 N Na_2_S_2_O_3_·5H_2_O using starch as an indicator until the blue color disappeared.

### Chemical analysis of the samples

All the investigated chemical parameters were determined according to the standard methods [21–24].

### Silicate determination

Sample solutions (maintained at 18–25 °C) were mixed with molybdate in 50 ml bottles and incubated for 10-minutes. A reducing reagent (metol/oxalic acid) was added to bring the volume to 50 ml, mixed, and incubated for 2–3 h. Absorbance was then measured at 810 nm using a spectrophotometer with a 1 cm path-length cuvette, using distilled water as the blank. Silicate concentrations were determined using a standard calibration curve prepared with sodium metasilicate (Na₂SiO₃·9 H₂O) in the concentration range 0.01–1.00 mg Si/L [25].

### Phosphate determination

30 ml sample aliquots were transferred to oxidation bottles, and 4 ml of oxidation reagent (ammonium molybdate and antimony potassium tartrate) was added to each, then the bottles were tightly sealed. Samples and reagent blanks (reagent + distilled water) were boiled for 30 min at 110–115 °C. After cooling to room temperature, samples were swirled to dissolve any precipitate, then transferred to measuring cylinders, adjusted to 35 ml with distilled water, and mixed. For analysis, 25 ml of each oxidized sample was mixed with 2 ml of ascorbic acid in a test tube to remove residual chlorine. 8 ml of acid-molybdate reagent was then added, and after 5 min, absorbance was measured at 880 nm using a 1 cm cuvette with distilled water as a reference. Phosphate concentrations were determined using a calibration curve prepared from potassium dihydrogen phosphate (KH₂PO₄), with a concentration range 0.01–1.00 mg PO₄-P/L [25].

### Ammonia determination

Samples were analyzed within 3 h of collection. For each analysis, 25 mL of sample was mixed with 1 mL citrate buffer, 1 ml of reagent A (0.71 µmol NH₃-N/mL), and 1 ml of reagent B (0.071 µmol NH₃-N/ml) in a glass bottle. Samples were then stoppered and incubated in the dark for 2–6 h (or overnight). Absorbance was measured at 630 nm, using distilled water as the reference. Ammonia concentrations were determined using a calibration curve prepared from standard ammonium chloride (NH_4_Cl) solutions in the range 0–1.0 mg/l NH₃-N.

### Nitrate determination

To 100 ml of each sample in an Erlenmeyer flask, 2.0 ml of concentrated ammonium chloride was added and mixed. The solution was then passed through a reduction column. The initial 40 ml of eluate was discarded, and the subsequent 50 ml was collected for analysis. After reduction, 1.0 ml of sulfanilamide solution (10 g/l in 1.5 M HCl) was added and allowed to react for 2–8 min, followed by the addition of 1.0 ml of N-(1-naphthyl)-ethylenediamine dihydrochloride solution (1 g/l). After color development for 10 min to 2 h, a pink to reddish-purple azo dye was formed, indicating the presence of nitrite (converted from nitrate during reduction). Absorbance was measured at 540 nm using a 1 cm path-length cuvette, with distilled water as the reference. Nitrate concentrations were determined from a standard curve (0–1.0 mg/l NO₃⁻-N) prepared using potassium nitrate solutions [26].

### Bacterial isolation and preservation

Water samples from El-Max and Abu Qir, along with wastewater from fish and oil factories, were serially diluted in 0.9% NaCl to obtain dilutions from 10⁻¹ to 10⁻⁷. A 50 µl of each dilution were spread onto LB agar plates (yeast extract, peptone, NaCl, and agar at 5,10,10 and 18 g/l, respectively) and incubated at 30 °C for 24–48 h. Resulting pure colonies were then individually transferred to fresh LB agar plates to confirm purity. Purified bacterial colonies were preserved using two methods: short-term preservation on LB agar slants at 4 °C, and long-term preservation in cryotubes containing 750 µl sterile 60% glycerol and 250 µl LB broth at -20 °C [27].

### Small scale design of MFC

#### Anodic chamber

The anodic chambers were constructed from sterile 50 ml conical centrifuge tubes. The screw cap of each tube was perforated with sterile needles to create a small opening for the titanium wire and a slightly larger opening for the agar bridge. After assembling the anodic chambers, each was filled with 20 ml of inoculated LB broth derived from the respective waste sample and then brought to a final volume of 50 mL with sterile LB broth [16, 28].

#### Cathodic chamber

Cathodic chambers were similarly prepared using sterile 50 mL conical centrifuge tubes with perforated caps for the titanium wire and agar bridge. Each chamber was filled with 50 mL of tap water [16].

#### Electrodes, wires, and resistors

Graphite bars (6 × 3 × 1 cm) sourced from battery waste were used as anode and cathode electrodes in each MFC. Each graphite bar was connected to a titanium wire using elastic bands, with the wire exiting the anodic or cathodic chamber through the small hole in the cap. The wires from both electrodes were then connected through a 1000 Ω resistor. The 1000 Ω external resistance was chosen as a standard value in MFC studies that approximates typical internal resistances, facilitating optimal power transfer during initial screening without overshoot. This value balances electron flux and microbial adaptation, as lower resistances can limit biofilm formation while higher ones reduce current. Also the 1000 Ω resistor was selected for its role in ensuring the circuit operated within a safe and measurable range for components and instrumentation [16].

#### Agar bridge

The agar bridge of each MFC was prepared by injecting 3% hot agar solution into rubber tubes (10 cm length each). After the solidification of the agar, each edge of the rubber tube was embedded into either the anodic or the cathodic liquids to allow the hydrogen protons to move from the anodic chambers to the cathodic chambers [16].

#### Measurements

**Fig. 1:**
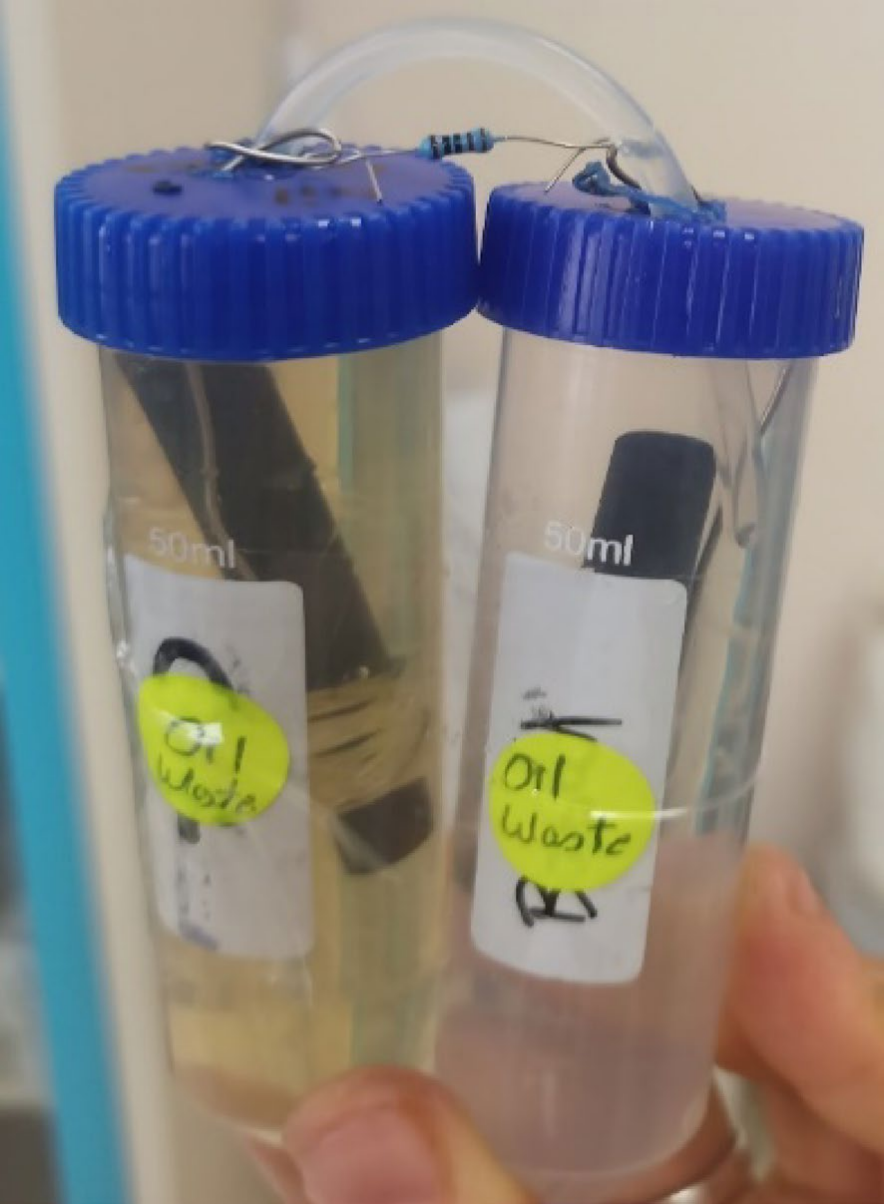
A representative photo of the MFC with all components after construction

All designed MFCs were incubated at 25 °C for several days. The voltage output from each cell was measured periodically after incubation, with the resistor disconnected each measurement [16]. A digital multimeter (UNI-T UT61E, Uni-Trend Technology, China) was used to record the voltage. A representative image of the complete MFC setup, showing all components post-assembly, is provided in Fig. 1.

#### Selection of high voltage-producing bacterial consortia

Based on the initial MFC experiments, four samples showing the highest voltage out-put (El-Max surface, Abu-Qir bottom, Oil factory waste water, and Fish factory waste water) were selected for further analysis. From each sample, individual bacterial strains were isolated and purified to evaluate their electricity-generating potential independently using the two-chamber MFC system [29].

#### MFC setup for individual bacterial isolates

The same MFC configuration and components were used to evaluate the electricity-generating performance of each individual bacterial isolate. For each test, a 20% (v/v) inoculum of the respective isolate was introduced into the anodic chamber at the start of the experiment. In the initial experimental round, three isolates from the El-Max surface water sample and four isolates from the fish factory wastewater sample were tested. In the second round, four isolates from the oil factory wastewater sample and three isolates from the Abu Qir bottom water sample were evaluated.

### Molecular identification of high voltage-producing bacterial strains

The most efficient voltage-producing bacterial strains were identified through *16 S rRNA* gene sequencing. Genomic DNA was extracted from pure bacterial cultures, and the *16 S rRNA* gene was amplified via PCR using universal primers. The PCR products were purified and sequenced using Sanger sequencing. Resulting sequences were analyzed using the BLAST tool against the NCBI GenBank database. Bacterial identification was determined based on sequence similarity (≥ 97% for species-level identification), and corresponding GenBank accession numbers were recorded for future reference [30]. Additionally, the phylogenetic placement of the isolates was determined based on the alignment of the obtained 16 S rRNA sequences, and a phylogenetic tree was constructed using MEGA7 software.

#### Optimizing of MFC anolyte composition using a Plackett-Burman experimental design

To evaluate the relative influence of six experimental variables (pH, inoculum size, oxygen, mediator and resistance) on MFC performance, a Plackett-Burman design (PBD) was employed, with modifications adapted from Abdel-Fattah et al. [16]. Each variable was tested at two levels: low (− 1) and high (+ 1). The experimental matrix consisted of eight independent trials; each conducted in 50 mL anolyte volumes within MFC chambers. Voltage output was measured using a multimeter and used as the response variable.

The Plackett-Burman design follows the first-order linear model:

$$Y\,=\,{\beta _0}\,+\,\sum\limits_{{i=1}}^{K} {{\beta _i}{X_i}} $$

where Y is the measured voltage response, β₀ is the model intercept, β**i** represents the effect coefficient of the variable, and x_i_ is the coded level of that variable. A Pareto chart was constructed to visualize the absolute effect of each variable on voltage output, indicating their relative significance regardless of direction (positive or negative).

## Results and discussion

### Physical parameters analysis

The physical characteristics of the sampling locations and waste streams (Table 1) reveal markedly distinct environmental conditions that are likely to influence the indigenous microbial community composition and activity, and, consequently, the performance of MFCs [31]. The relatively consistent temperatures of the water samples (24.5–25.5 °C) suggest stable conditions conducive to a broad range of mesophilic microorganisms. In contrast, the significantly elevated temperature of the oil factory waste (72 °C) is indicative of industrial processing, which may select for thermotolerant microbial population, but could also inhibit the activity of mesophilic electrogens, thereby potentially affecting MFC performance [32]. The salinity measurements highlight the diverse nature of the sampled environments. As expected, the Abu Qir (38.41–38.91 g/l) and El-Max (30.66–30.70 g/l) water samples exhibited typical seawater salinity levels. The extremely high salinity of the Abu Qir sediment (4700 g/l) compared to the El-Max sediment (8.011 g/l) likely reflects differences in evaporation rates, freshwater inputs, and sediment composition at these locations. Such hyperhaline conditions are known to impose strong selective pressures, favoring halotolerant or halophilic microbial communities [33]. Similarly, industrial wastewaters showed considerable variation in salinity. The fish factory wastewater exhibited a salinity of 560 g/l, while the oil factory wastewater was even more saline at 1140 g/l. These elevated salinity levels, while lower than those of the Abu Qir sediment, are still significant enough to influence microbial selection and, consequently, the nature and efficiency of MFC microbial consortia. The pH measurements revealed a range of conditions from slightly alkaline in the water samples (7.85–7.92) to slightly acidic in the fish factory waste (6.76) and highly alkaline in the oil factory waste (11.78). This extreme alkalinity likely results from the use of caustic chemicals during oil extraction processes. These pH variations are critical, as pH is a key factor influencing microbial enzyme activity and overall metabolic function. The highly alkaline conditions may restrict microbial diversity, favoring only those species capable of tolerating high pH, thus potentially constraining MFC performance due to limited functional microbial populations [34]. Lastly, dissolved oxygen (DO) concentrations in the water samples were relatively high, ranging from 19.24 to 23.92 mg/l, indicating well-oxygenated conditions favorable for aerobic microbial processes. Overall, the observed physicochemical variability across sites—particularly in temperature, salinity, and pH—highlights the selective pressures acting on microbial communities in each habitat. These environmental factors are critical in shaping microbial composition and, by extension, their electrogenic potential when applied in MFC systems.

**Table 1:** Physical parameters of seawater and industrial wastewater samples

Samples/physical parameters	Temperature (^o^C)	Salinity (g/l)	Conductivity (mS/cm)	pH	DO (mg/l)
Abu Qir surface water sample	25.5	38.41	59.1	7.89	20.8
Abu Qir bottom water sample	25	38.91	58.7	7.92	19.24
El-max surface water sample	25	30.7	47.73	7.9	23.92
El-max bottom water sample	24.5	30.66	47.44	7.85	20.28
Abu Qir sediment sample	27.1	4700	9420	7.8	-
El-max sediment sample	26.5	8.011	16.8	7.4	-
Fish factory waste	23	560	865	6.76	-
Oil factory waste	72	1140	1983	11.78	-

### Chemical parameters analysis

The nutrients analysis revealed stark differences in ammonia, phosphate, silicate, nitrite, nitrate, and total suspended matter (TSM) concentrations across the sampling locations, indicative of different levels of organic pollution and nutrients enrichment [35]. As shown in Table 2, Abu Qir water exhibited the lowest ammonia levels (17.5–50.4 µg/l), suggesting relatively low levels of recent organic matter input and decomposition. El-Max water showed higher ammonia concentrations (456.4–466.9 µg/l), potentially reflecting greater anthropogenic influence from urban runoff or sewage discharge [36]. Strikingly, the industrial wastes displayed extremely elevated ammonia levels (Fish: 1,275,346.944 µg/l; Oil: 20,139,853,824 µg/l), indicating substantial ongoing organic matter breakdown and ammonification processes within these waste streams. These high ammonia concentrations could be both a readily available nitrogen source for microbial growth and a potential inhibitor of certain electrogenic bacteria at sufficiently high concentrations [37]. Phosphate concentrations followed a similar trend to ammonia, with low levels in Abu Qir water (~ 3 µg/l), higher levels in El-Max water (157,728 − 165,168 µg/l), and orders of magnitude higher concentrations in the oil factory waste (16,735 − 656,000 µg/l). The high phosphate levels in the oil waste likely reflect the presence of phosphate-based detergents or other additives used in industrial processes. While phosphate is an essential nutrient for microbial growth, excessively high concentrations can disrupt microbial community balance and potentially inhibit certain metabolic pathways [38]. Silicate concentrations were relatively consistent in Abu Qir water (119.7–122.892 µg/l), increased in El-Max water, and elevated in the industrial wastes. This suggests a potential contribution from industrial processes or geological sources. Elevated nitrite and nitrate levels in El-Max water and the industrial wastes likely reflect nitrification processes and/or direct inputs from industrial discharges or agricultural runoff. The TSM values, which reflect the particulate load in the samples, mirrored the trends observed for ammonia and phosphate. The extremely high TSM values found in the industrial waste samples indicate a substantial load of particulate organic matter associated with these discharges. This particulate matter could serve as a source of organic carbon for microbial degradation [39, 40].

**Table 2:** Chemical parameters analysis of seawater and industrial wastewater samples

Sample	Ammonia (µg/l)	PO_4_ (µg/l)	SiO_4_ (µg/l)	NO_2_ (µg/l)	NO_3_ (µg/l)	T.S.M. (mg/l)
Abu Qir surface water	17.5	2.976	122.892	6.65	35.28	0.01427
Abu Qir bottom water	50.4	2.976	119.7	6.3	43.12	0.01438
El-max surface water	466.9	165.168	1155.504	274.05	490	0.01466
El-max bottom water	456.4	157.728	924.084	260.05	476.642	0.01416
Fish factory wastewater	-	1,275,346.944	3,632,336.4	11,882.5	47,941.6	734.5
Oil factory wastewater	-	20,139,853,824	16,735,656	293,632.5	412,109.6	1443

**Table 3:** Voltage outputs from microbial fuel cells (MFCs) inoculated with individual microbial isolates: (A) isolates derived from El-Max surface seawater and fish factory wastewater; (B) isolates derived from oil factory wastewater and Abu-Qir bottom sediment

(A) Isolate code	Time (h)						
	0	48	72	120	144	168	264
Max S1	00.3 mV	0.229 V	0.287 V	0.292 V	0.270 V	0.271 V	0.269 V
Max S2	00.1 mV	0.376 V	0.390 V	0.416 V	0.197 V	0.224 V	0.178 V
Max S3	00.5 mV	0.340 V	0.358 V	0.331 V	0.340 V	0.337 V	0.319 V
Fish GH1	109.5 mV	0.187 V	0.182 V	0.297 V	0.244 V	0.257 V	0.189 V
Fish GH2	69.6 mV	0.173 V	0.240 V	0.164 V	0.133 V	0.166 V	0.147 V
Fish GH3	09.8 mV	0.240 V	0.334 V	0.305 V	0.292 V	0.393 V	0.237 V
Fish 4	12.5 mV	0.254 V	0. 259 V	0.265 V	0.339 V	0.172 V	0.303 V

(B) Isolate code	Time (h)					
	0	120	168	192	288	336
Oil 3	00.4 mV	0.281 V	0.280 V	0.432 V	0.542 V	0.147 V
Oil A	81.4 mV	0.004 V	0.283 V	0.290 V	0.162 V	0.279 V
Oil GHO5	2.2 mV	0.002 V	0.362 V	0.291 V	0.243 V	0.325 V
Oil GHO7	174 mV	0.236 V	0.228 V	0214 V	0.295 V	0.209 V
QB1	120 mV	0.384 V	0.212 V	0.363 V	0.210 V	0.466 V
QB2	127 mV	0.285 V	0.266 V	0.204 V	0.339 V	0.278 V
QB	163 mV	0.431 V	0.452 V	0.465 V	0.453 V	0.386 V

### Electricity generation from wastewater using enriched microbial consortia

**Fig. 2:**
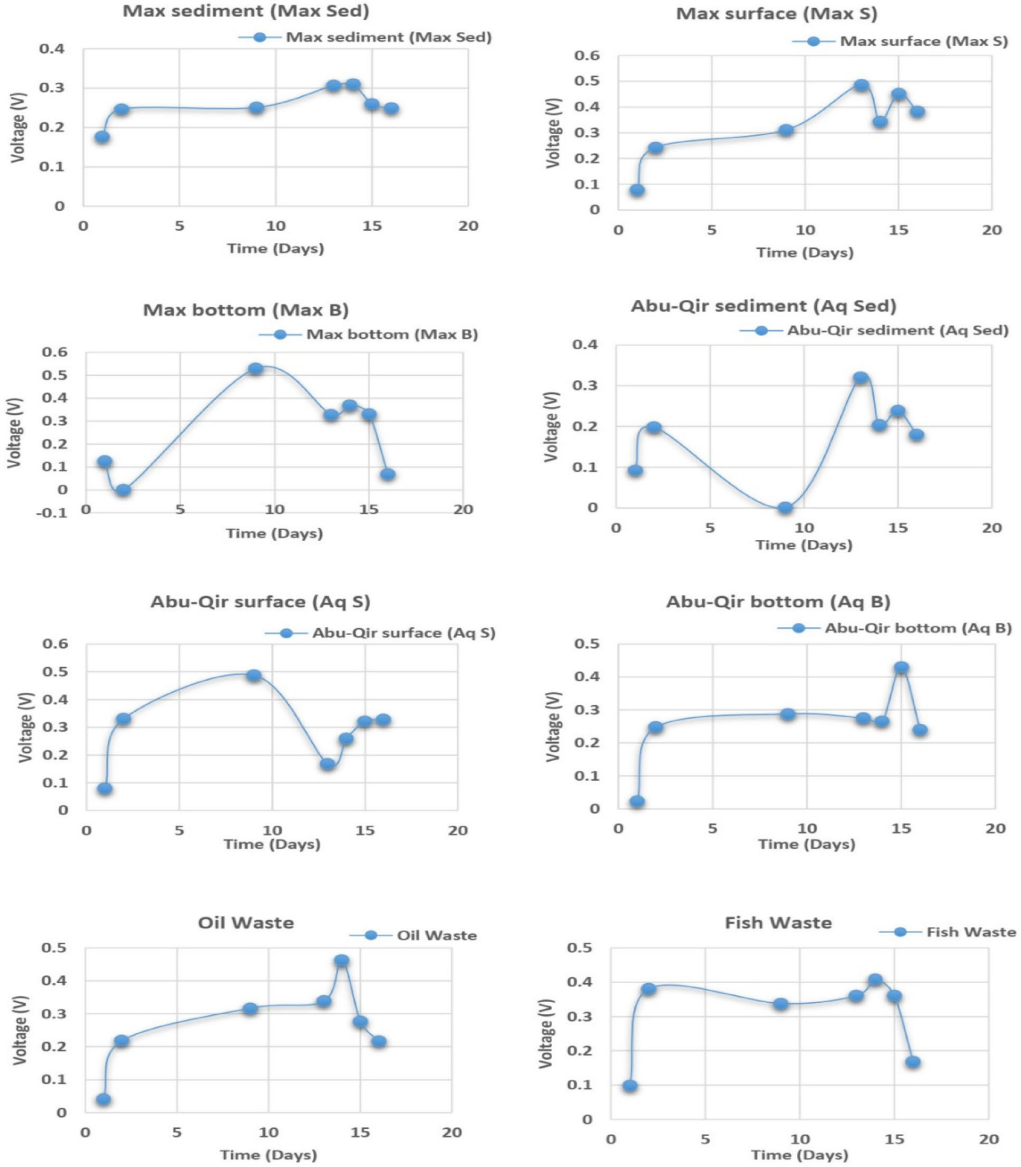
Voltage outputs from microbial fuel cells (MFCs) inoculated with microbial consortia derived from collected seawater and industrial wastewater samples over time, STD = ± 0.5

Screening of microbial consortia in MFCs revealed varying bioelectricity-generating potential across diverse environmental samples [41]. After 24-hour of incubation, voltage outputs ranged from 21.9 mV (Abu-Qir bottom) to 179.8 mV (Max sediment), indicating early differences in microbial activity and electron transfer efficiency among the samples (Fig. 2 and Table 1S). Notably, the El-Max bottom consortium also demonstrated strong early performance with 124.7 mV, suggesting the presence of rapidly adapting, metabolically active electrogens.

A consistent trend was observed where voltage outputs increased over time, reaching a peak around 312 h, followed by a gradual decline (Fig. 2 and Table 1S). Notably, some samples deviated from this pattern: for instance, the Abu Qir bottom sample reached its maximum output of 0.430 V at 360 h, while the El-Max bottom consortium peaked earlier at 216 h with 0.530 V. These variations highlight the distinct metabolic dynamics and electron transfer strategies among microbial communities from different environments. Most voltage outputs fell within the range of 0.2 to 0.4 V, indicating moderate but sustained electrogenic capacity across the tested consortia. The performance differences observed can be attributed to several factors, including variations in microbial community structure, substrate availability, salinity, and pH, all of which influence extracellular electron transfer (EET) processes [42, 43]. For example, the high early voltage in the El-Max bottom sample may result from a microbial population capable of rapid colonization and efficient electron release to the anode. In contrast, the delayed peak in the Abu Qir bottom sample suggests a slower adaptation phase or differences in biofilm development and maturation [44]. Importantly, consortia from fish factory wastewater and oil factory wastewater also showed relatively stable voltage profiles throughout the experimental period, despite the chemical complexity of their environments. This suggests a degree of resilience and adaptability among wastewater-derived electrogens, making them attractive candidates for real-world MFC applications [45]. Based on both maximum voltage output and long-term stability [40], four consortia—El-Max surface, Abu Qir bottom, fish factory wastewater, and oil factory wastewater—were selected for further investigation. These consortia (highlighted in yellow in Table 1S) demonstrated the most promising electrogenic profiles in terms of output magnitude and consistency over time. The selection was guided by the need to identify microbial communities capable of sustained bioelectricity generation, a key criterion for their potential application in scalable and efficient MFC systems.

### Bacterial isolation and purification

**Fig. 3:**
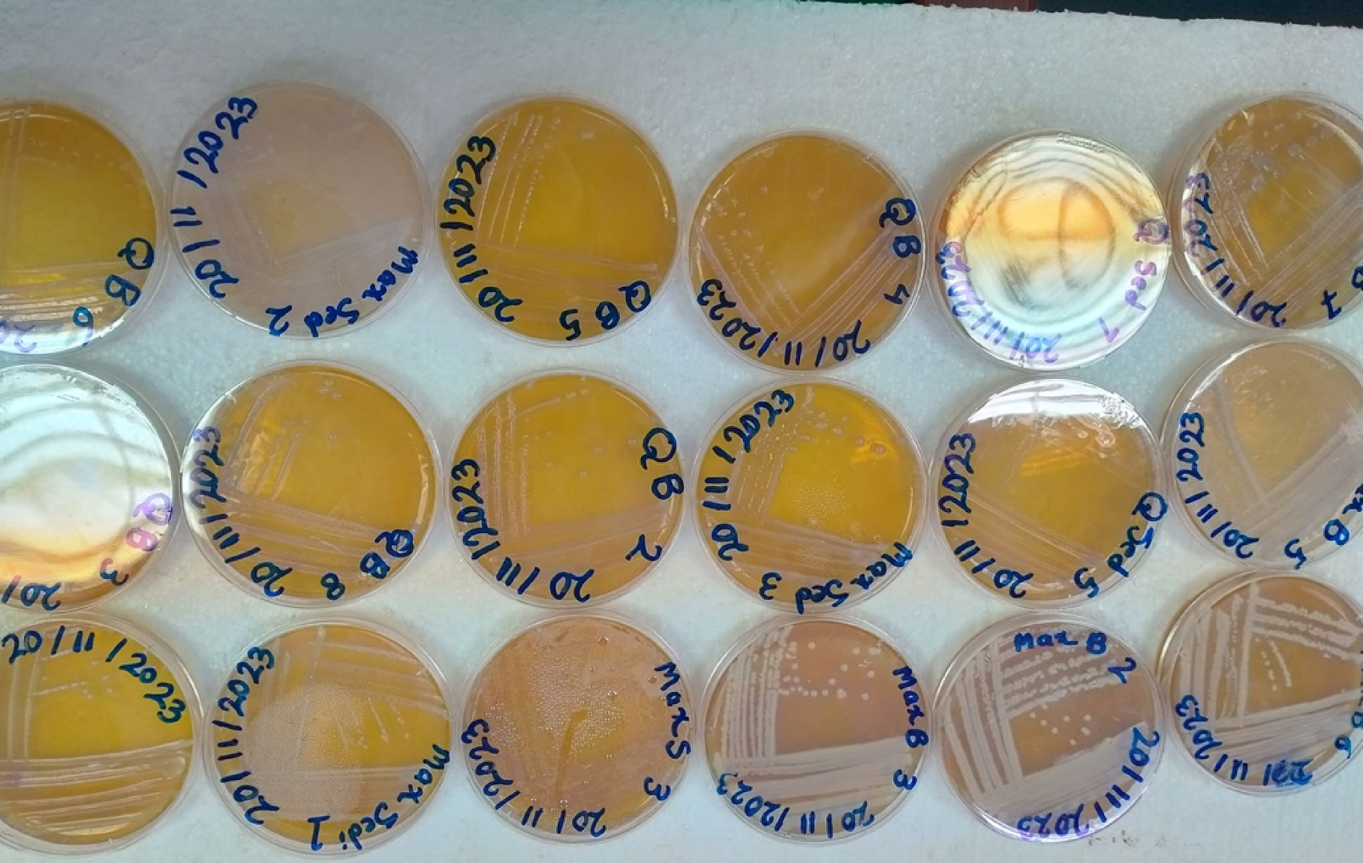
Some representative photos of the isolated and purified bacterial isolates from the Mediterranean and Borg El-Arab water samples on LB agar plates

A total of 59 bacterial strains were successfully isolated from diverse environmental sources, including El-Max surface seawater, oil factory wastewater, Abu Qir bottom seawater, and fish factory wastewater as depicted in Fig. 3. This marks a significant advancement in the current phase of the study, which focuses on identifying individual bacterial strains with electrogenic potential. The substantial number of isolates reflects the rich microbial diversity inherent to these environments [46], as previously evidenced by the differing electricity-generating capacities of their corresponding microbial consortia in MFC systems [47]. Earlier tests with enriched consortia showed distinct voltage outputs (0.430–0.530 V), confirming active electrogens and highlighting the adaptability of wastewater-derived communities under electrochemical stress. Building on these findings, the current phase involves the isolation and purification of individual strains, enabling a more targeted approach for screening electrogenic potential. The present focus is to evaluate the electricity-generating capabilities of each isolate within controlled, single-strain MFC setups. This approach is expected to clarify the specific contributions of individual strains to the consortial performance observed previously. Given the wide physicochemical variation among the original sampling environments, including differences in salinity, pH, and organic load, these isolates are likely to exhibit diverse metabolic traits and extracellular electron transfer efficiencies. This screening step is essential for identifying robust, high-performing electrogens with potential for application in scalable and efficient MFC systems.

### Electrogenic potential of individual bacterial isolates from the collected samples

To evaluate the electrogenic capabilities of individual bacterial strains, isolates from four environmental sources were inoculated separately into single-chamber MFCs. The first screening phase included three isolates from El-Max surface water (Max S1, Max S2, Max S3) and four from fish factory wastewater (Fish GH1, Fish GH2, Fish GH3, and Fish 4). Initial voltage outputs ranged from 0.3 to 109.5 mV (Table 3A & Fig. 1S), with notable increases observed after 48 h, reaching between 0.173 V and 0.376 V—indicative of active microbial metabolism and adaptation to the anodic environment. Among these, Max S2 demonstrated the highest electrogenic performance, achieving a peak voltage of 0.416 V at 120 h, suggesting efficient extracellular electron transfer (EET) and robust biofilm formation. Other isolates, such as Fish GH3 and Fish 4, also produced stable outputs (ranging from 0.2 to 0.3 V), albeit with minor fluctuations, indicating variability in their electron transfer stability. Based on voltage magnitude and consistency, Max S2 and Fish GH3 were selected for further study. In the second screening phase, individual isolates from oil factory wastewater (Oil 3, Oil A, Oil GH05, and Oil GH07) and Abu-Qir bottom sediment (QB1, QB2, QB) were tested. As shown in Table 4B and Fig. 1S, all isolates exhibited electrogenic activity to varying degrees. Oil 3 produced the highest voltage output among all tested strains, peaking at 0.542 V after 288 h, indicating strong electrogenic potential and long-term metabolic stability. Oil A reached a lower peak of 0.290 V at 192 h, yet still demonstrated consistent performance. Among the Abu-Qir isolates, QB showed notably stable voltage production (~ 0.4 V) over a broad timeframe (120–288 h), with a slight decline to 0.386 V at 336 h, suggesting a well-developed biofilm and sustained electron transfer capacity. Based on these observations, Oil 3 and QB were selected for further study. Across both screening phases, the selection of isolates was based on two key criteria: (1) the magnitude of voltage output and (2) the ability to maintain stable performance over time. These selected isolates represent a diverse pool of electrogenic bacteria capable of functioning in variable environmental and physicochemical conditions, such as those present in industrial and marine wastewaters.

### Bacterial identification of the selected isolates

**Fig. 4:**
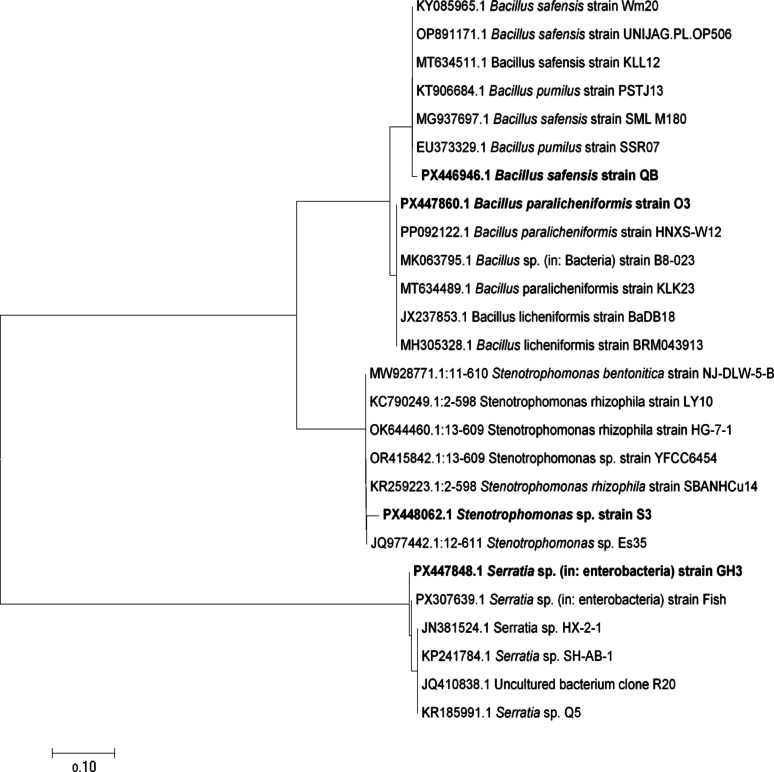
Phylogenetic tree of the selected bacterial isolates based on 16 S rRNA sequences. Neighbor-joining tree showing the relationship between the four isolates (shown in bold) and their closest GenBank reference strains

The identification of bacterial isolates via *16 S rRNA* gene sequencing has provided valuable insight into the microbial diversity contributing to voltage generation in MFCs. The isolates characterized in this study—*Stenotrophomonas* sp. strain S2 (El-Max surface seawater, AC: PX448062), *Bacillus paralicheniformis* strain O3 (Oil factory wastewater, AC: PX447860), *Bacillus safensis* strain QB (Abu-Qir bottom seawater, AC: PX446946), and *Serratia* sp. strain GH3 (Fish wastewater, AC: PX447848) have been demonstrated promising electrochemical potential and underscore the diverse origins of electrogenic bacteria in marine and industrial environments. A phylogenetic tree showing the relationship between the isolates and their closest relatives was constructed based on aligned 16 S rRNA sequences and presented in Fig. 4. Traditionally, several bacterial genera such as *Geobacter*, *Shewanella*, and *Pseudomonas* have been widely studied and utilized in MFCs due to their well-established mechanisms of extracellular electron transfer (EET) and efficient biofilm formation on anodes. For instance, *Geobacter sulfurreducens* is often considered as a model electrogen, known for its conductive pili and direct electron transfer mechanisms [42, 48]. Similarly, *Shewanella oneidensis* is recognized for its versatile metabolism and ability to transfer electrons via soluble redox mediators [43]. These canonical electrogens have laid the foundation for MFC research and performance benchmarking. In contrast, the bacterial strains isolated in this study represent non-canonical or underexplored electrogens, yet they exhibit comparable or potentially synergistic EET capabilities. Members of the genus *Stenotrophomonas*, for example, are not traditionally associated with high electrogenic activity. However, recent studies suggest that certain strains possess redox-active compounds and biofilm-forming abilities, supporting their participation in EET processes [49, 50]. This could explain their effective role in enhancing voltage generation within the MFC setup. *Bacillus paralicheniformis* has been previously reported for its robust biofilm formation and resilience under harsh industrial conditions [51]. Its metabolic versatility and stress tolerance likely support stable and consistent electron transfer, making it suitable for treating high-strength wastewaters while simultaneously generating bioelectricity. These characteristics align with other Bacillus species known for their durable cell walls and diverse respiration strategies, contributing to their relevance in bioelectrochemical systems. Likewise, *Bacillus safensis*, notable for its extreme environmental tolerance and endospore-forming capacity, is well-adapted for use in MFCs. Its identification in the present study reinforces the potential of *Bacillus* species as core contributors to electrogenic consortia. Their thick peptidoglycan layers and adaptive metabolic pathways are thought to facilitate strong electrochemical interaction with the MFC anode surface [52]. The isolation of *Serratia* sp. strain GH3 from fish processing wastewater adds another layer of functional diversity to the electrogenic microbial community. *Serratia* species, particularly *S. marcescens*, are facultative anaerobes capable of utilizing various carbon sources and producing redox-active secondary metabolites such as prodigiosin, which may assist in EET mechanisms [53]. Their adaptability to aquatic environments further supports their performance in MFCs fed with fish industry effluents. Collectively, the identification of these strains from diverse ecological niches highlights the untapped potential of naturally occurring bacteria in enhancing MFC performance. While model organisms like *Geobacter* and *Shewanella* continue to set benchmarks, the discovery of novel or underexplored electrogens such as *Stenotrophomonas*, *Bacillus*, and *Serratia* species broadens the horizon for future MFC development. Their successful application in voltage generation demonstrates that bioprospecting in varied environments can yield high-performing electrogenic candidates. Moreover, the ecological adaptability and metabolic diversity of these isolates contribute not only to electricity production but also to bioremediation of contaminated environments. This dual functionality aligns with the overarching goals of MFC technology—to integrate sustainable energy generation with effective wastewater treatment, supporting the transition to a circular, bio-based economy [54].

### Optimization of bioelectricity production using the Plackett–Burman design

To identify the most influential parameters affecting bioelectricity production, the Plackett–Burman Design (PBD) was applied to evaluate six operational factors: resistance, inoculum size, time, oxygen, pH, and mediator. The effect of these variables on voltage output was assessed for four bacterial strains: *Stenotrophomonas* sp. S2, *Bacillus paralicheniformis* O3, *Bacillus safensis* QB, and *Serratia* sp. GH3. The derived regression model describing the relationship between the input variables and voltage output in case of *Stenotrophomonas* sp. S2 is as follows:

$$ \begin{aligned} {\mathrm{Y}}{\mkern 1mu} & = {\mkern 1mu} 0.{\mathrm{3395}}{\mkern 1mu} - {\mkern 1mu} 0.0{\mathrm{413X}}_{{\mathrm{1}}} {\mkern 1mu} - {\mkern 1mu} 0.{\mathrm{1195X}}_{{\mathrm{2}}} {\mkern 1mu} \\ & + {\mkern 1mu} 0.0{\mathrm{653X}}_{{\mathrm{3}}} {\mkern 1mu} - {\mkern 1mu} 0.00{\mathrm{33X}}_{{\mathrm{4}}} {\mkern 1mu} + {\mkern 1mu} 0.{\mathrm{1487X}}_{{\mathrm{5}}} {\mkern 1mu} + {\mkern 1mu} 0.0{\mathrm{8}}0{\mathrm{3X}}_{{\mathrm{6}}} \\ \end{aligned} $$

**Fig. 5:**
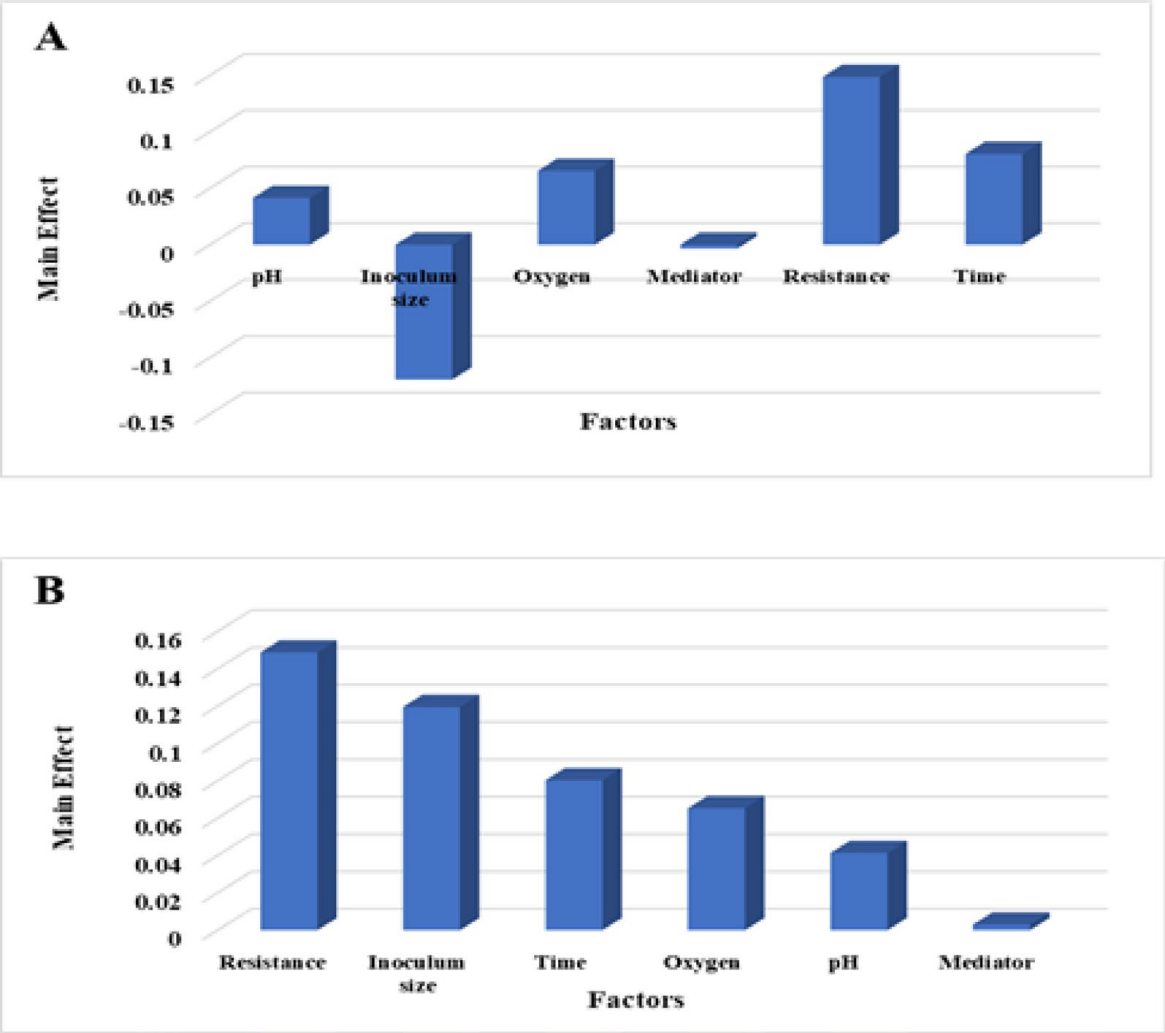
Main effects chart showing the influence of each factor on voltage production by *Stenotrophomonas* sp. S2 (**A**). Pareto chart illustrating the relative significance of each factor affecting voltage production by *Stenotrophomonas* sp. S2 (**B**)

Where Y represents the predicted voltage, and X₁ to X₆ correspond to pH, inoculum size, oxygen, mediator, resistance, and time, respectively. Voltage outputs across the eight experimental trials ranged from 0.170 V (Trial 7) to 0.525 V (Trial 3), measured after three days of incubation (Table 4). The main effect analysis (Fig. 5A) indicated that all six factors influenced electricity generation to varying degrees, ranked by impact as follows: Resistance >Inoculum size >Time >Oxygen >pH >Mediator. Positive regression coefficients for resistance, time, pH, oxygen, and mediator suggest that increasing these variables within the tested range enhanced voltage output. Notably, the highest coefficient was observed for resistance (+ 0.1487), highlighting it as the most influential factor in maximizing bioelectricity by S2 strain. In contrast, the negative coefficient for inoculum size (-0.1195) implies that lower inoculum densities may promote improved electron transfer efficiency, potentially by reducing intra-population competition for substrates and minimizing oxygen diffusion limitations [16].

**Table 4:** Optimization of anolyte solution in a microbial fuel cell (MFC] using statistical experimental design (PBD), with individual testing of *Stenotrophomonas* sp. strain S2, *Bacillus paralicheniformis* strain O3, *Bacillus safensis* strain QB and *Serratia* sp. strain GH3

Trials	pH	Inoculum size (%)	Oxygen	Mediator	Resistance (Ω)	Time (Days)	Response voltage output (V) by strain			
							S2	O3	OB	GH3
1	-1(6.5)	1(40)	1(+)	-1(Nil)	1(1000)	1(6)	0.468	0.215	0.329	0.211
2	-1(6.5)	-1(20)	1(+)	1(+)	-1(100)	1(6)	0.403	0.343	0.352	0.31
3	1(7.5)	-1(20)	-1(Nil)	1(+)	1(1000)	-1(3)	0.525	0.207	0.181	0.31
4	1(7.5)	1(40)	-1(Nil)	-1(Nil)	1(1000)	1(6)	0.420	0.298	0.277	0.503
5	-1(6.5)	1(40)	1(+)	-1(Nil)	-1(100)	1(6)	0.204	0.39	0.279	0.263
6	1(7.5)	-1(20)	1(+)	1(+)	-1(100)	-1(3)	0.325	0.44	0.222	0.401
7	-1(6.5)	1(40)	-1(Nil)	1(+)	1(1000)	-1(3)	0.170	0.203	0.259	0.175
8	1(7.5)	-1(20)	-1(Nil)	-1(Nil)	-1(100)	-1(3)	0.201	0.418	0.21	0.195
Model summary and statistical parameters in case of S2 strain										
Coefficients	0.041333	-0.1195	0.065333	-0.00333	0.148667	0.080333	-			
Main effect	0.082666	-0.239	0.130666	-0.00666	0.297334	0.160666	-			
* P-*.value	0.329932	0.150897	0.220421	0.910561	0.100099	0.181689	-			
t-stat	1.753625	-4.1396	2.771859	-0.14142	6.307392	3.408255	-			
* R*^*2*^ Model	0.98									
Model summary and statistical parameters in case of Q3 strain										
* Coefficients*	0.0495	0.037	0.008	0.003	-0.098	0.001	-			
Main effect	0.099	0.074	0.016	0.006	-0.196	0.002	-			
* P-*.value	0.185538	0.290838	0.685406	0.873092	0.095734	0.957192	-			
* t-stat*	3.333503	2.034472	0.538748	0.202031	-6.59966	0.067344	-			
* R*^*2*^ Mode	0.99									
Model summary and statistical parameters in case of QB strain										
* Coefficients*	-0.0955	-0.06138	-0.06463	-0.02975	0.020125	0.069875	-			
Main effect	-0.191	-0.12276	-0.12926	-0.0595	0.04025	0.13975	-			
* P-*.value	0.178966	0.231705	0.220987	0.328681	0.362841	0.116204	-			
* t-stat*	-3.46301	-2.62514	-2.76415	-1.76166	1.560315	5.417491	-			
* R*^*2*^Model	0.98									
Model summary and statistical parameters in case of GH3 strain										
Coefficients	0.152833	0.079	0.006833	0.096167	-0.03233	0.107333				
Main effect	0.305666	0.158	0.013666	0.192334	-0.06466	0.214666				
* P-*.value	0.037266	0.0878371	0.5850985	0.0591224	0.1720368	0.0530015				
t-stat	17.0636	7.201685	0.762931	10.73686	-3.60997	11.9836				
* R*^*2*^ Model	0.99									

**Fig. 6:**
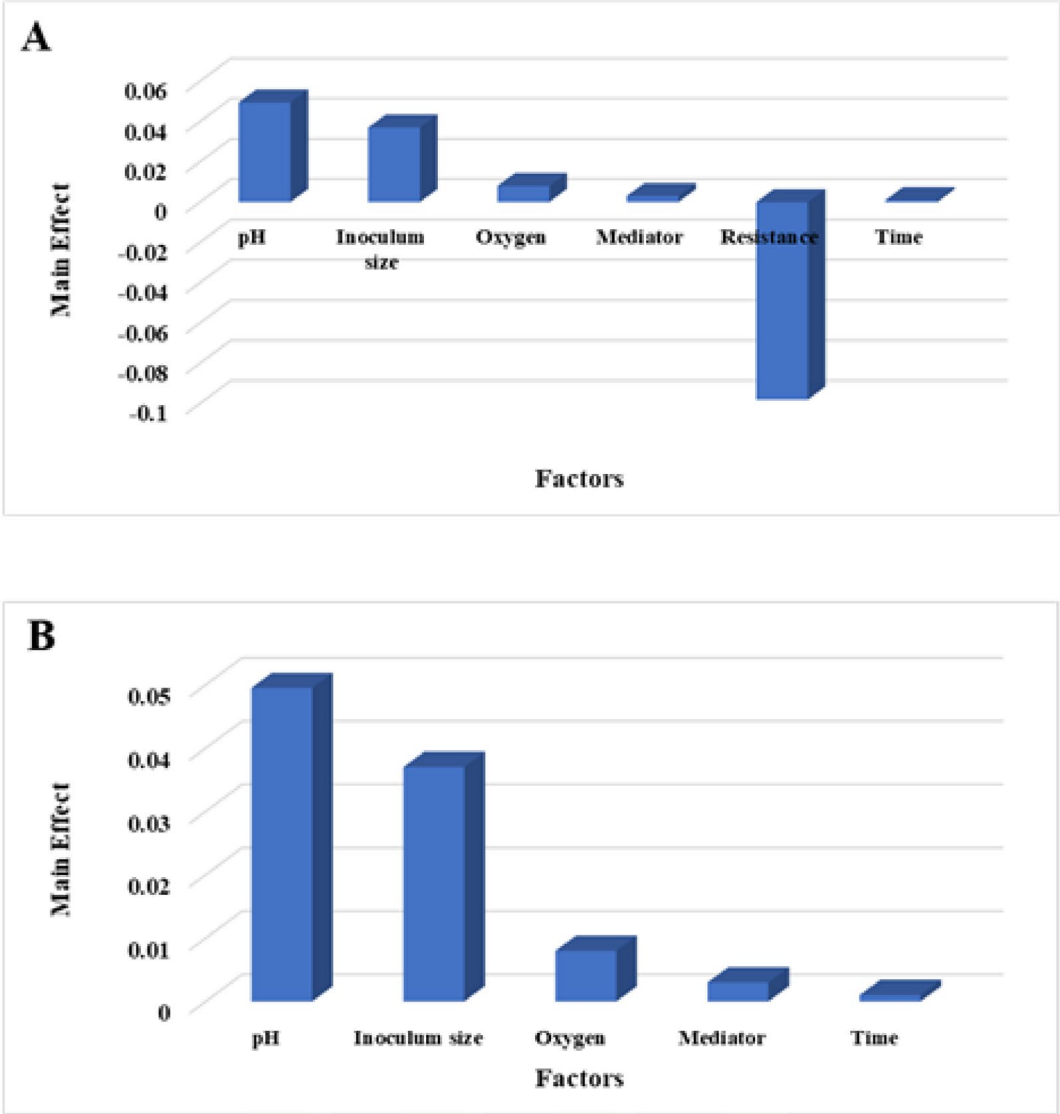
Main effects chart showing the influence of each factor on voltage production by *Bacillus paralicheniformis* O3 (**A**). Pareto chart illustrating the relative significance of each factor affecting voltage production by *Bacillus paralicheniformis* O3 (**B**)

The influence of pH, oxygen, and mediators aligns with existing literature, which reports that maintaining a neutral to slightly alkaline pH, the presence of moderate oxygen levels, and the addition of electron shuttles (e.g., humic acids or phenazines) can enhance MFC performance by supporting microbial metabolism and extracellular electron transfer pathways [55–57]. The Pareto chart (Fig. 5B) visually reinforced the significance of these factors, providing a clear representation of their relative impact. The resistance and inoculum size appearing well above the significance threshold, underscoring their dominant influence. A *t-test* was used to assess the statistical confidence of each variable, with effects considered significant at a confidence level of ≥ 80% (*p* ≤ 0.2), adding statistical rigor to the analysis. The model demonstrated excellent predictive accuracy, as indicated by a coefficient of determination (*R²* = 0.98), confirming a strong correlation between observed and predicted values (Table 4). This high *R²* value substantiates the reliability and robustness of the PBD model [16] in optimizing operational conditions for electricity generation using *Stenotrophomonas* sp. S2. The relationship between the studied variables and the voltage output was captured by the following regression model for *Bacillus paralicheniformis* O3 based on Plackett-Burman Design (PBD):

$$\begin{aligned} {\mathrm{Y}}\, & =\,0.{\mathrm{31425}}\,+\,0.0{\mathrm{495}}{{\mathrm{X}}_{\mathrm{1}}}\,+\,0.0{\mathrm{37}}{{\mathrm{X}}_{\mathrm{2}}}\, \\ & +\,0.00{\mathrm{8}}{{\mathrm{X}}_{\mathrm{3}}}\,+\,0.00{\mathrm{3}}{{\mathrm{X}}_{\mathrm{4}}} - \,0.0{\mathrm{98}}{{\mathrm{X}}_{\mathrm{5}}}\,+\,0.00{\mathrm{1}}{{\mathrm{X}}_{\mathrm{6}}} \\ \end{aligned} $$

where Y denotes the predicted voltage, and X₁ to X₆ correspond to pH, inoculum size, oxygen, mediator, resistance, and time, respectively. Voltage outputs across the experimental trials ranged from 0.203 V (Trial 7) to 0.440 V (Trial 6), as shown in Table 4. The main effect analysis (Fig. 6A) identified resistance as the most influential factor, followed by pH, inoculum size, oxygen, mediator, and time. The positive correlations observed for time, pH, oxygen, mediator, and inoculum size suggest that increasing these factors (within the tested ranges) generally leads to higher voltage. This aligns with the understanding that these factors contribute to favorable conditions for microbial growth, metabolic activity, and electron transfer. Conversely, the negative correlation for resistance suggests that lower resistance values promote higher voltage, likely due to improved electron flow and reduced energy losses within the MFC. These results align with studies demonstrating that factors like electrolyte concentration, stirring, NaCl addition, and inoculum characteristics significantly influence bioelectricity generation. Optimizing these parameters can increase power output and accelerate electricity production [16, 57].

The Pareto chart (Fig. 6B) visually reinforced the relative importance of each variable, providing a clear and intuitive representation of their impact on bioelectricity generation. This visual confirmation enhances the understanding of the key drivers of MFC performance. Statistical significance was rigorously determined using a t-test, with effects considered significant at confidence levels ≥ 80% (*p* ≤ 0.2), ensuring the reliability of the identified factors. The model demonstrated a high degree of accuracy, with a determination coefficient (*R2* = 0.99; Table 4) signifying a strong relationship between predicted and observed voltage. This high *R2* value validates the model’s predictive capability for power generation and confirms its usefulness for optimizing MFC operation [16].

The model captured the linear effects of six variables—pH, time, oxygen, inoculum size, mediator, and resistance—on voltage output for *Bacillus safensis* QB, was expressed as:

$$ \begin{aligned} {\mathrm{Y}}{\mkern 1mu} & = \,{\mkern 1mu} 0.{\mathrm{23975}} - 0.0{\mathrm{955X}}_{{\mathrm{1}}} - {\mkern 1mu} 0.0{\mathrm{6138X}}_{{\mathrm{2}}} \\ & - {\text{ }}0.0{\mathrm{6463X}}_{{\mathrm{3}}} - {\mkern 1mu} 0.0{\mathrm{2975X}}_{{\mathrm{4}}} {\mkern 1mu} + \,{\mkern 1mu} 0.0{\mathrm{2}}0{\mathrm{125X}}_{{\mathrm{5}}} {\mkern 1mu} \\ & + {\mkern 1mu} 0.0{\mathrm{69875X}}_{{\mathrm{6}}} \\ \end{aligned} $$

Here, Y is the predicted voltage, while X₁ to X₆ correspond to pH, time, oxygen, inoculum size, mediator, and resistance, respectively.

**Fig. 7:**
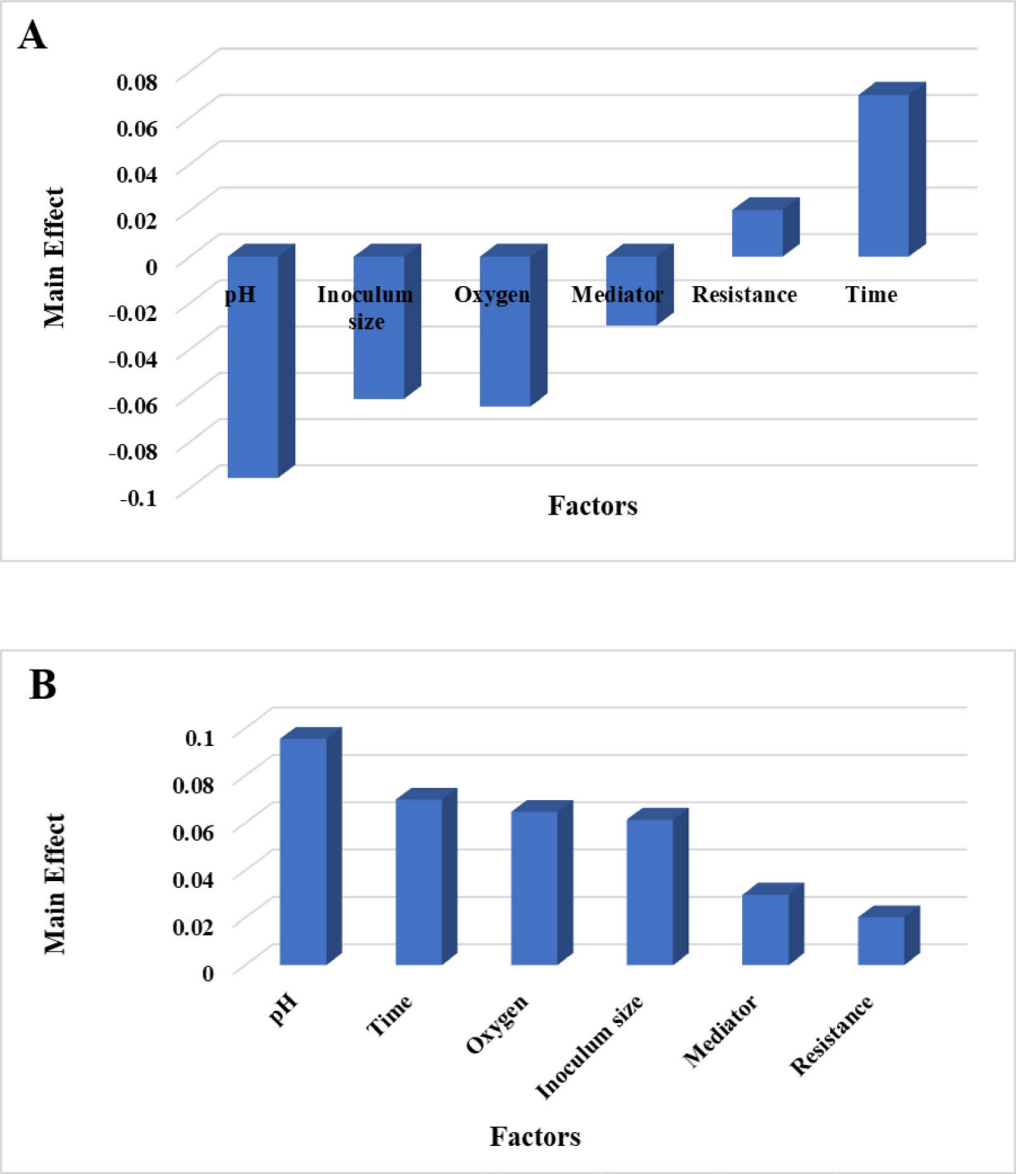
Main effects chart showing the influence of each factor on voltage production by *Bacillus safensis* QB (**A**). Pareto chart illustrating the relative significance of each factor affecting voltage production by *Bacillus safensis* QB (**B**)

The PBD trials yielded voltage outputs ranging from 0.181 V (Trial 3) to 0.352 V (Trial 2) (Table 4). Main effect analysis (Fig. 7A) revealed that pH was the most influential factor, followed by time, oxygen, inoculum size, mediator, and resistance. This descending order of influence highlights pH as a key lever for enhancing bioelectricity production in *Bacillus safensis* QB-based systems. This signifies that adjusting pH has the most potential to affect voltage output, followed by manipulating time, oxygen levels, and so forth. The positive correlation between resistance and time with voltage implies that, within the tested ranges, increasing these factors tends to enhance power generation. This could be due to improved electron flow at higher resistances (up to a certain point) or increased metabolic activity with longer incubation times. Conversely, the negative correlation observed for pH, inoculum size, oxygen, and mediator suggests that lower values of these factors approach optimum conditions for maximizing bioelectricity output. For example, a lower inoculum size might reduce competition for resources, leading to more efficient electron transfer. Similarly, lower pH values could favor specific metabolic pathways that enhance power generation. These findings are in line with a wide range of research demonstrating that the efficiency and power output of bioelectricity production systems, including MFCs and other bioelectrochemical platforms, are fundamentally governed by environmental and operational parameters such as the type of substrate used, the characteristics of the electrode material, and the specific composition of the microbial community involved [58, 59]. The Pareto chart (Fig. 7B) visually validated the relative importance of each factor, offering a clear and easily interpretable representation of their influence. The statistical significance of each variable effect was rigorously assessed using a t-test, with effects considered significant at confidence levels near or above 80% (*p* ≤ 0.2), reinforcing the reliability of the findings. The high determination coefficient (*R2* = 0.98; Table 4) confirmed a strong correlation between the predicted and observed voltage values, thus validating the model’s reliability for predicting power generation and enabling informed decision-making for MFC optimization. The modeled response for bioelectricity production by *Serratia* sp. GH3, based on the Plackett-Burman design (PBD), was expressed as:

$$ \begin{aligned} {\mathrm{Y}} & = \,{\mkern 1mu} 0.{\mathrm{296}}{\mkern 1mu} \, + \,{\mkern 1mu} 0.{\mathrm{152833X}}_{{\mathrm{1}}} {\mkern 1mu} + {\mkern 1mu} \,0.0{\mathrm{79X}}_{{\mathrm{2}}} \\ & + \,{\mkern 1mu} 0.00{\mathrm{6833X}}_{{\mathrm{3}}} {\mkern 1mu} + {\mkern 1mu} 0.0{\mathrm{96167X4}} - 0.0{\mathrm{3233X}}_{{\mathrm{5}}} {\mkern 1mu} \\ & + {\mkern 1mu} \,0.{\mathrm{1}}0{\mathrm{7333X}}_{{\mathrm{6}}} \\ \end{aligned} $$

where Y represents the predicted voltage output.

**Fig. 8:**
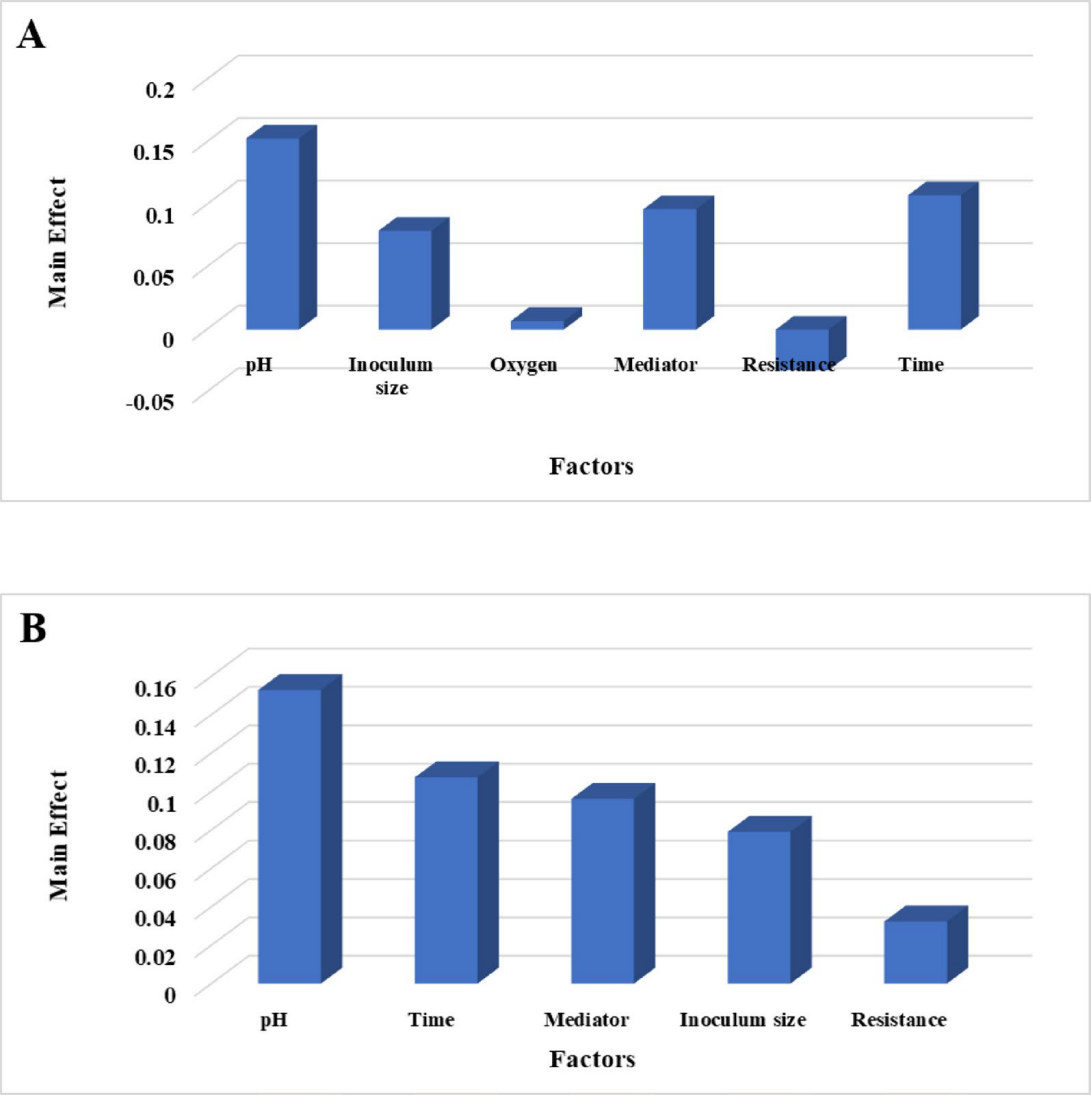
Main effects chart showing the influence of each factor on voltage production by *Serratia* sp. GH3 (**A**). Pareto chart illustrating the relative significance of each factor affecting voltage production by *Serratia* sp. GH3 (**B**)

**Fig. 9:**
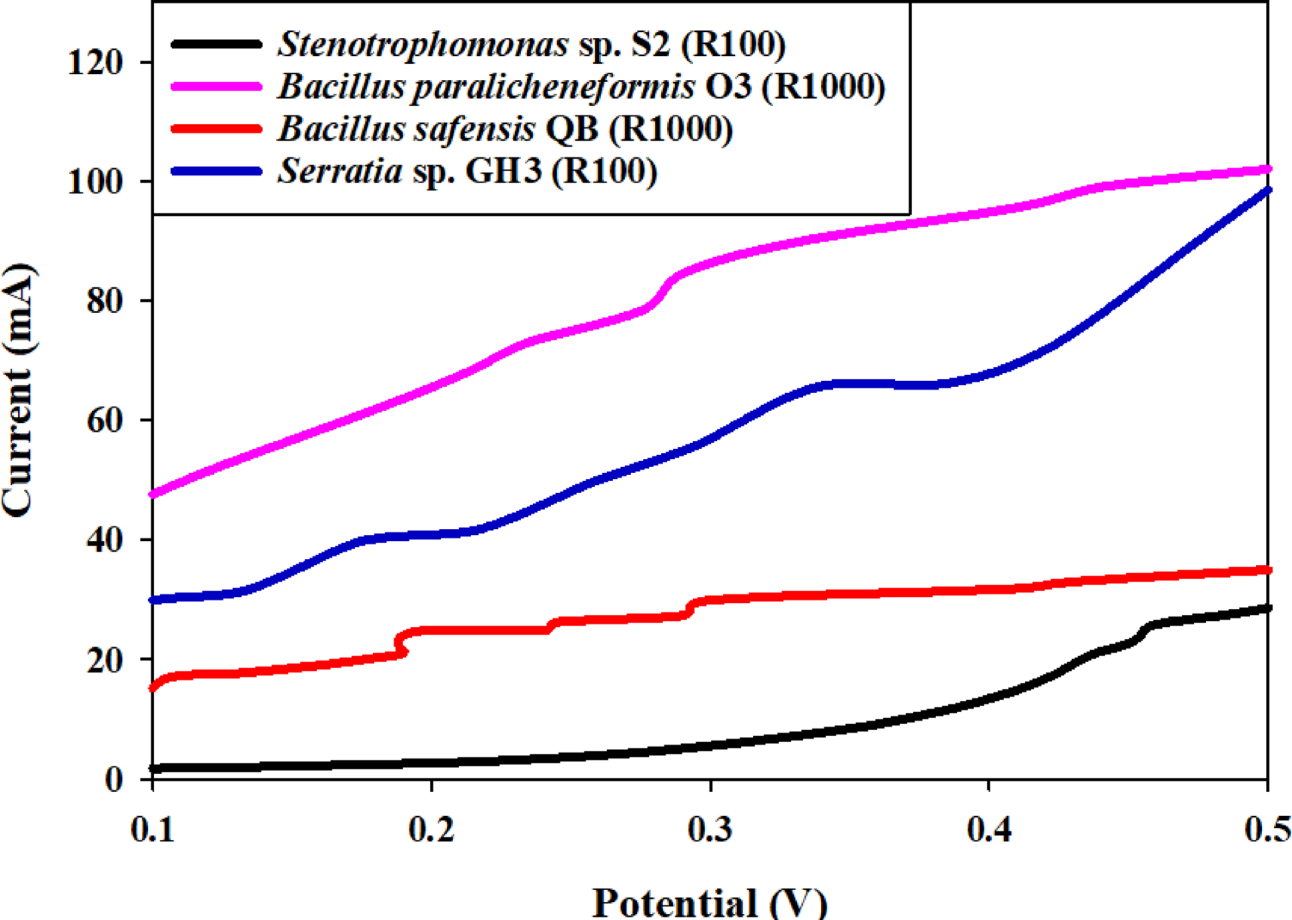
Voltage-Current (V-I) analysis and electrochemical response of four isolates

Voltage outputs observed in the PBD experiments ranged from 0.175 V (Trial 7) to 0.503 V (Trial 4) (Table 4). Main effect analysis (Fig. 8A) identified the six tested factors—pH, time, mediator, inoculum size, resistance, and oxygen—as influential variables affecting bioelectricity production, ranked in order of decreasing importance. This ranking suggests that pH has the most significant impact on voltage output, followed by time, mediator concentration, and the other factors. The positive effects of pH, time, oxygen, mediator, and inoculum size on voltage generation indicate that, within the tested ranges, increasing these variables enhances bioelectricity production. This finding suggests that these factors play a role in promoting favorable conditions for microbial growth, electron transfer, or overall metabolic efficiency. In contrast, the negative effect of resistance suggests that lower resistance values are closer to optimal conditions, potentially due to enhanced electron flow and reduced energy dissipation within the MFC circuit. Similar optimization studies have identified key variables such as substrate concentration, stirring, NaCl addition, and inoculum size as significant for maximizing voltage output, and have shown that factors like stirring and substrate concentration positively affect bioelectricity generation, while higher resistance can reduce output due to less efficient electron flow [16, 60]. The positive influence of factors like pH, oxygen, and inoculum size on voltage is consistent with findings that these parameters promote microbial activity and electron transfer, while lower resistance is generally favorable for efficient energy conversion in MFCs [60]. The Pareto chart (Fig. 8B) visually reinforces the relative importance of each factor, offering a clear and intuitive understanding of their individual contributions to the overall bioelectricity generation process. This visual validation strengthens the reliability of the identified key drivers of MFC performance. A rigorous *t-test* confirmed the statistical significance of each variable (*p* ≤ 0.2, ≥ 80% confidence levels), lending further support to the identified key influencers. The model’s high determination coefficient (*R2* = 0.99; Table 4 demonstrates a strong correlation between predicted and observed voltage values, validating its accuracy and predictive capabilities for bioelectricity generation. These findings suggest that carefully balancing these influential factors, increasing time, pH, oxygen, mediator and inoculum size while decreasing resistance, is critical for optimizing MFC performance [16]. Based on the previously presented Plackett–Burman Design (PBD) data for the four tested strains (*Stenotrophomonas* sp. S2, *Bacillus paralicheniformis* O3, *Bacillus safensis* QB, and *Serratia* sp. GH3), the collective findings are summarized in Table 5, which provides a comparative summary of the voltage outputs and factor rankings for all four tested strains on bioelectricity production. While resistance, pH, and **i**noculum size emerged as common influential parameters across the strains, the direction and magnitude of their effects varied. For instance, resistance enhanced voltage in *Stenotrophomonas* sp. S2 but reduced output in *B. paralicheniformis* O3 and *Serratia* sp. GH3. Similarly, pH showed strain-specific effects depending on microbial physiology. These findings demonstrate the strain-dependent nature of operational parameter optimization and highlight the importance of customized approaches when designing MFC systems. The high predictive accuracy of the models (*R²* = 0.98–0.99) confirms the effectiveness of PBD in identifying critical factors for maximizing bioelectricity production. The analysis using the PBD models confirmed that resistance, pH, and inoculum size are the most influential operational factors affecting bioelectricity production across the four tested microbial strains (R_2_ values of 0.98–0.99 validate the models’ reliability). However, the optimal conditions are highly strain-specific, necessitating customized optimization: for instance, while Resistance was the dominant positive factor for *Stenotrophomonas* sp. S2, it was a negative influence for *Bacillus paralicheniformis* O3, highlighting a difference in internal resistance matching. Similarly, pH was the primary determinant for *Bacillus safensis* QB (negative effect, favoring lower pH) and *Serratia* sp. GH3 (positive effect, favoring higher pH). These results confirm the microbial-dependent nature of MFC operation and demonstrate that factors like low inoculum size can sometimes enhance electron transfer efficiency by reducing intra-population competition. The efficient and sustainable application of microbial fuel cells (MFCs) relies not only on maximizing peak power output but, critically, on maintaining a stable voltage for extended operational periods. Our current study demonstrated that the isolated bacterial strains sustained a stable and measurable voltage output for a continuous period of 15 days, a significant finding when compared to many published reports that primarily focus on maximum voltage or power density over very short testing windows (e.g., 24 to 72 h) or show a rapid decline shortly after the initial peak due to factors like biofilm detachment, substrate depletion, or material degradation [61, 62]. This short-lived performance, while sometimes achieving high initial values, limits the practical application of MFCs. The consistent voltage production observed in our research suggests that our isolates possess key characteristics for long-term viability, including a robust and resilient electroactive biofilm, efficient and sustained substrate utilization, and a high tolerance to micro environmental changes, all of which are essential for reliable, scaled-up MFC designs and contrast favorably with studies interested only in the transient, low-time voltage production in MFC research. Table 6 indicated the significant differences between low-cost laboratory MFCs and commercial-scale systems and also provides an effective illustration of our low-cost system’s advantages and limitations [63]. In MFC voltage increases with current contradicts the fundamental behavior of an MFC acting as a power source, where voltage always decreases due to internal energy losses as current is drawn. However, for comparative analysis, the strains are ordered from best electrochemical performance (highest sustained voltage at increasing current) to worst: *Stenotrophomonas* sp. S2 >*Bacillus paralicheniformis* O3 >*Bacillus safensis* QB >*Serratia* sp. GH3 indicated in Fig. 9.

**Table 5:** Comparison of strain responses

Strain	Isolation source	Max voltage (V)	Top influential factors	External resistance Ω	*R*² value
*Stenotrophomonas* sp. S2	El-Max surface seawater	0.525	Res., Inoc*., Time	1000	0.98
*Bacillus paralicheniformis* O3	Oil factory wastewater	0.440	Res., pH, Inoc*	100	0.98
*Bacillus safensis* QB	Abu-Qir bottom seawater	0.352	pH, Time, Oxygen	1000	0.99
*Serratia* sp. GH3	Fish factory wastewater	0.503	pH, Time, Mediator	100	0.99

^*^"Inoc.” denotes the inoculum size, “Positive” corresponds to 1000 Ω, while “Negative” corresponds to 100 Ω

**Table 6:** Comparison of low-cost laboratory MFCs and commercial-scale microbial fuel cell systems

Feature	Low cost lab setup	Commercial scale systems
Cost	5$-10$ per unit	735$ − 36,000$ (Per reactor volume)
Key components	Graphite from batteries, agar bridges, tap water cathode	Specialized electrodes, membranes, advanced materials
Electrochemical output	Small (mW scale)	Higher (W/m^3^scale)
Primary value / application	High cost-effectiveness for screening and educational purposes	Economic viability via power generation & large-scale treatment
Scalability for viability	Requires significant design improvement	Ready for industrial/pilot scale

## Conclusion

This study successfully demonstrated the potential of MFCs to generate bioelectricity from diverse wastewater sources. Bacterial consortia and individual isolates were obtained from four distinct environments: Max surface seawater, fish factory wastewater, oil factory wastewater, and Abu-Qir bottom seawater. These sources yielded electrogenic microorganisms capable of converting organic matter into electricity. Initial screening of microbial consortia revealed significant variations in voltage generation, emphasizing the influence of environmental conditions on microbial community composition and activity. Further isolation and individual testing identified four promising electrogenic strains with enhanced bioelectricity-generating capabilities: *Stenotrophomonas* sp. S2 (from Max seawater), *Bacillus paralicheniformis* O3 (from oil factory wastewater), *Bacillus safensis* QB (from Abu-Qir seawater), and *Serratia* sp. GH3 (from fish factory wastewater). To optimize power generation, the Plackett–Burman Design (PBD) was employed to evaluate the influence of six key operational parameters: pH, time, oxygen, inoculum size, mediator, and resistance. While the relative impact and directionality of these variables differed among strains, all were found to significantly influence MFC performance. Main effect and Pareto analyses supported these findings, and the high determination coefficients *(R²* values ranging from 0.98 to 0.99) validated the predictive reliability of the developed models. The present work strongly validates the dual utility of wastewater-sourced electrogens for combined bioelectricity generation and treatment, successfully isolating and optimizing four promising strains from diverse saline and industrial effluents. The PBD modeling identified resistance, pH, and inoculum size as universally critical, yet highly strain-dependent, parameters, necessitating customized operational strategies for maximum efficiency. Specifically, the contrasting impact of resistance and pH across the strains (e.g., positive vs. negative effects) highlights that MFC design must be tailored to the specific microbial physiology to effectively harness these sustainable wastewater-derived resources. Ultimately, the high predictive accuracy of the models provides a robust framework for moving closer to a circular economy by optimizing these bioelectrochemical systems for simultaneous environmental remediation and bioenergy recovery. This work contributes meaningfully to the development of sustainable bioenergy solutions and supports progress toward a circular economy and cleaner energy future. Future research should focus on further characterizing the metabolic and electrochemical properties of the identified strains, exploring synergistic interactions within mixed microbial communities, and scaling up MFC systems for real-world applications.

## Supplementary Information

Below is the link to the electronic supplementary material.

Supplementary material 1.

## Data Availability

All data produced during this study are included in this published article.
