# Supplementary material for: Enhanced voltage generation in microbial fuel cells (MFCs) using bacterial isolates from seawater and industrial wastewater
Source: Microb Cell Fact. 2025 Dec 28;25:21. doi: 10.1186/s12934-025-02892-w (PMC12836919; doi:10.1186/s12934-025-02892-w)
Supplement: Supplementary file 1 — Supplementary material 1. [file 12934_2025_2892_MOESM1_ESM.docx]

**Enhanced voltage generation in microbial fuel cells (MFCs) using bacterial isolates from seawater and industrial wastewater**

### ****Table (1S):** Voltage output (V) from Microbial Fuel Cells (MFCs) inoculated with microbial consortia from seawater and industrial wastewater samples.**

| **Sample** | **24 h** | **48 h** | **216 h** | **312 h** | **336 h** | **360 h** | **384 h** |
| --- | --- | --- | --- | --- | --- | --- | --- |
| Max sediment  (Max Sed) | 0.177 V | 0.247 V | 0.251 V | 0.307 V | 0.310 V | 0.260 V | 0.249 V |
| Max surface  (Max S) | 0.079 V | 0.244 V | 0.312 V | 0.486 V | 0.344 V | 0.453 V | 0.384 V |
| Max bottom  (Max B) | 0.125 V | 0.005 V | 0.530 V | 0.329 V | 0.369 V | 0.330 V | 0.070 V |
| Abu Qir sediment (Q Sed) | 0.092 V | 0.198 V | 0.001 V | 0.320 V | 0.204 V | 0.239 V | 0.180 V |
| Abu Qir surface  (Q S) | 0.079 V | 0.331 V | 0.488 V | 0.168 V | 0.259 V | 0.322 V | 0.327 V |
| Abu Qir bottom  (Q B) | 0.022 V | 0.248 V | 0.288 V | 0.275 V | 0.266 V | 0.430 V | 0.240 V |
| Oil factory wastewater | 0.041 V | 0.218 V | 0.317 V | 0.338 V | 0.462 V | 0.277 V | 0.217 V |
| Fish factory wastewater | 0.099 V | 0.381 V | 0.338 V | 0.361 V | 0.408 V | 0.361 V | 0.168 V |


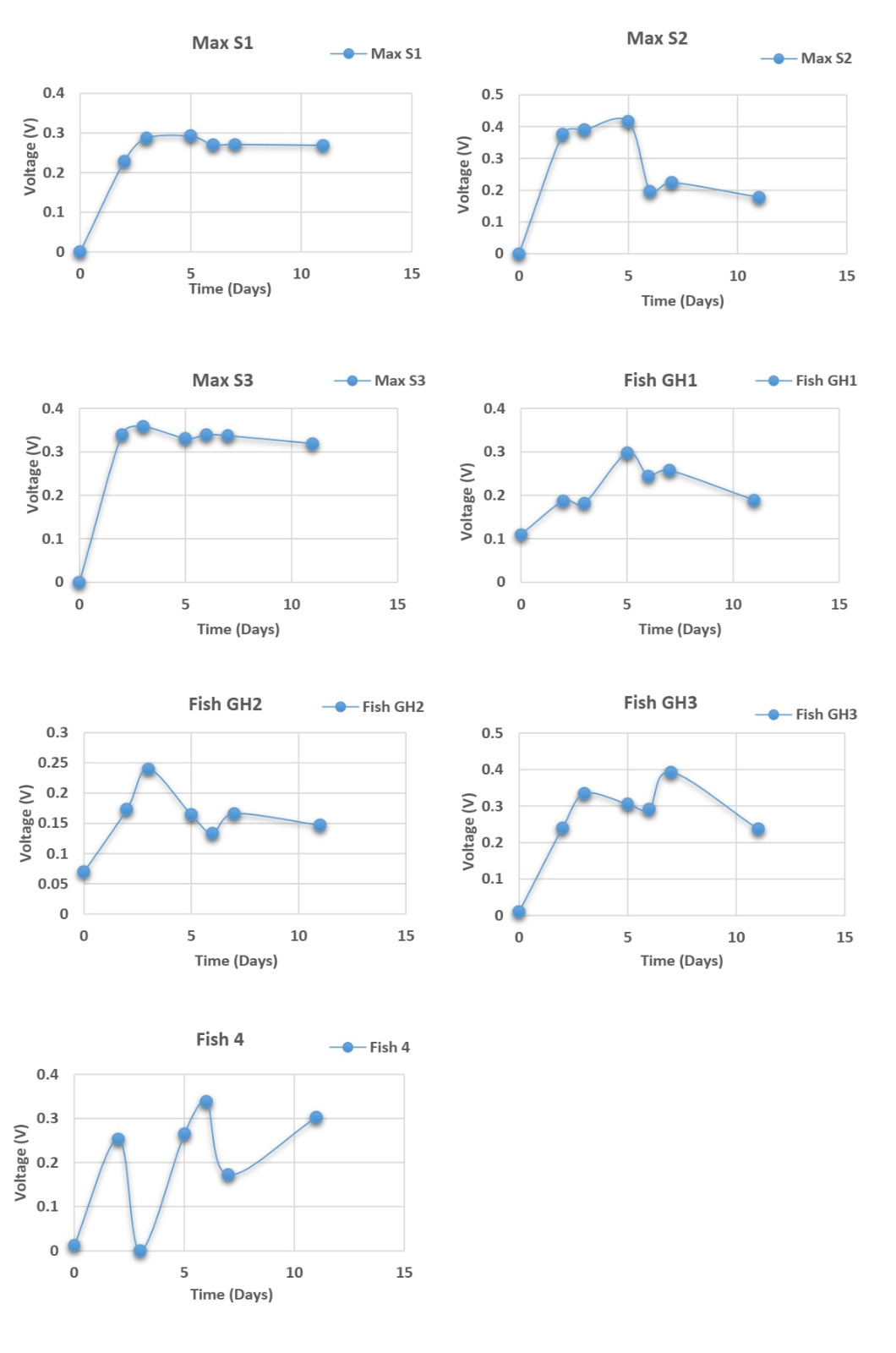


**A**


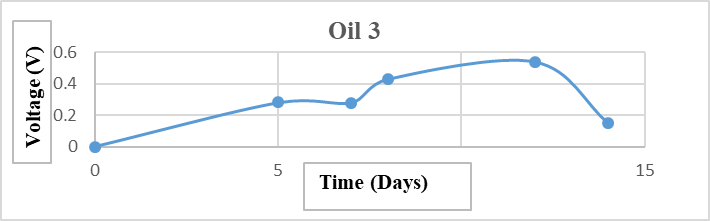

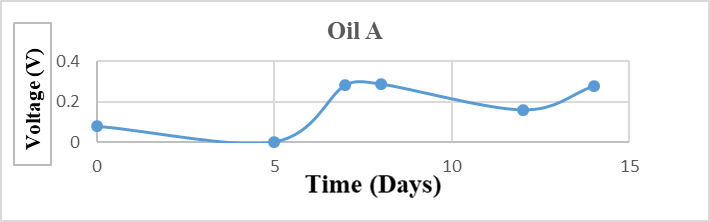

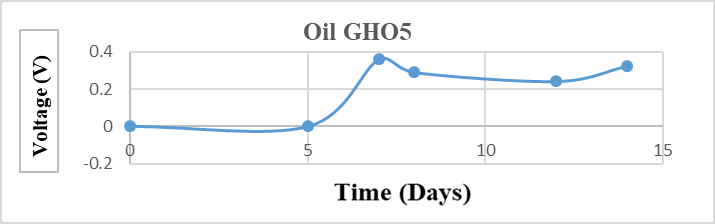

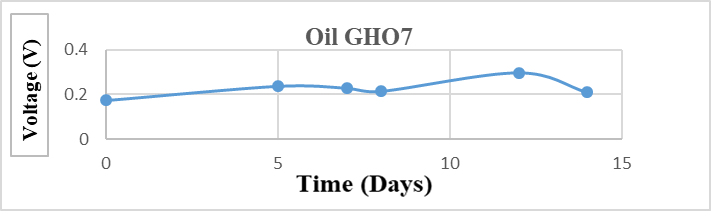

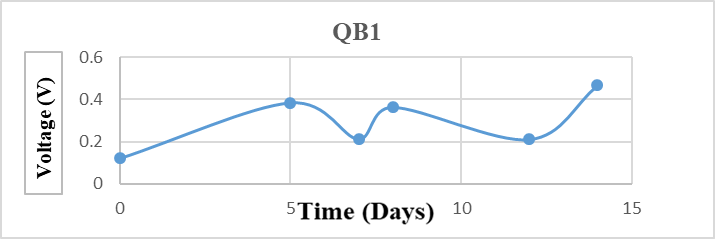

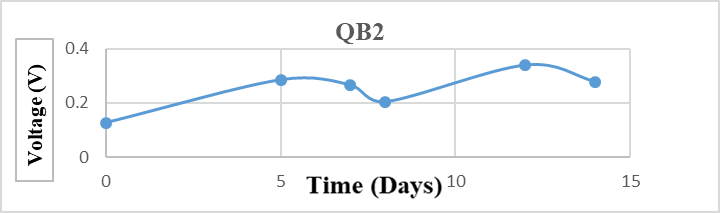

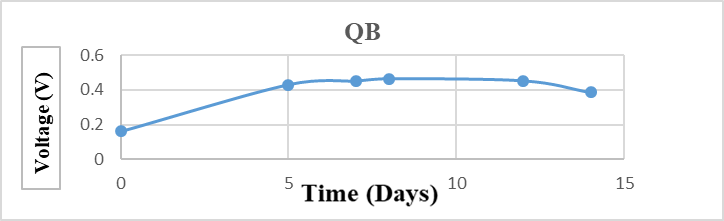


**B**

**Fig. (1S):** Voltage outputs from MFCs inoculated with individual microbial isolates: **(A)** isolates derived from El-Max surface seawater and fish factory wastewater; **(B)** isolates derived from oil factory wastewater and Abu-Qir bottom seawater, STD =±0.5.
